# Colony‐age‐dependent variation in cuticular hydrocarbon profiles in subterranean termite colonies

**DOI:** 10.1002/ece3.6669

**Published:** 2020-08-16

**Authors:** Johnalyn M. Gordon, Jan Šobotník, Thomas Chouvenc

**Affiliations:** ^1^ Entomology and Nematology Department Ft. Lauderdale Research and Education Center Institute of Food and Agricultural Sciences University of Florida Davie FL USA; ^2^ Faculty of Tropical AgriSciences CULS Prague Czech Republic

**Keywords:** chemical ecology, colony fusion, *Coptotermes gestroi*, recognition, social insects

## Abstract

Cuticular hydrocarbons (CHCs) have, in insects, important physiological and ecological functions, such as protection against desiccation and as semiochemicals in social taxa, including termites. CHCs are, in termites, known to vary qualitatively and/or quantitatively among species, populations, castes, or seasons. Changes to hydrocarbon profile composition have been linked to varying degrees of aggression between termite colonies, although the variability of results among studies suggests that additional factors might have been involved. One source of such variability may be colony age, as termite colony demographics significantly change over time, with different caste and instar compositions throughout the life of the colony. We here hypothesize that the intracolonial chemical profile heterogeneity would be high in incipient termite colonies but would homogenize over time as a colony ages and accumulates older workers in improved homeostatic conditions. We studied caste‐specific patterns of CHC profiles in *Coptotermes gestroi* colonies of four different age classes (6, 18, 30, and 42 months). The CHC profiles were variable among castes in the youngest colonies, but progressively converged toward a colony‐wide homogenized chemical profile. Young colonies had a less‐defined CHC identity, which implies a potentially high acceptance threshold for non‐nestmates conspecifics in young colonies. Our results also suggest that there was no selective pressure for an early‐defined colony CHC profile to evolve in termites, potentially allowing an incipient colony to merge nonagonistically with another conspecific incipient colony, with both colonies indirectly and passively avoiding mutual destruction as a result.

## Graphical abstract

Cuticular hydrocarbons play an important role in terms of nestmate recognition in eusocial insect taxa, including termites. The blend of these compounds is variable among castes in young termite colonies, but gradually convergences on a similar hydrocarbon blend in older colonies. Early heterogeneity in chemical profile and a subsequent inability to detect non‐nestmates may have explained previously observed ready fusion of incipient colonies.

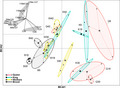

## 1 INTRODUCTION

Cuticular hydrocarbons (CHCs) are a major component of the wax layer of the insect outer epicuticle (Chapman, 2012) and have several recognized functions, such as protection from desiccation and disease, waterproofing, and individual labeling (Blomquist, Nelson, & Renobales, 1987; Dettner, 2014; Howard & Blomquist, 1982, 2005). CHCs are responsible for species recognition especially in social insects, and different species have qualitatively distinct CHC profiles, while the intraspecific variability consists of quantitative differences among the compounds forming the population‐ or colony‐specific blend (Howard & Blomquist, 1982, 2005). This function is employed by social parasites that are integrated into social insect colonies through chemical mimicry (Howard, McDaniel, & Blomquist, 1980; Howard et al., 1982; Lenoir, D'Ettorre, Errard, & Hefetz, 2001; van Zweden & D'Ettorre, 2010). Within intraspecific recognition, the CHC mixtures provide sufficient information to distinguish colony, caste, age, or reproductive status in eusocial insects (Clément, 1982; Bagnères, Rivière, & Clément, 1998; Clément & Bagnères, 1998; Funaro, Böröczky, Vargo, Schal, 2018; Howard et al., 1982; Howard & Blomquist, 2005; Singer, 1998). In termites, a blend of cuticular hydrocarbons, rather than any particular one, is responsible for caste recognition, with this blend comprised of both alkanes and alkenes, including methylalkanes (Haverty, Nelson, & Page, 1990; Howard & Blomquist, 1982, 2005; Howard, McDaniel, & Blomquist, 1978).

The role of CHCs in recognition and communication has been extensively studied in social Hymenoptera (Dani et al., 2005; Martin & Drijfhout, 2009; Singer, 1998; Vander Meer, Saliwanchik, & Lavine, 1989; van Zweden & D'Ettorre, 2010). In ants, it has been suggested that kin recognition is more the result of recognizing the dissimilar profiles of alien individuals rather than the similar profiles of nestmates (Guerrieri et al., 2009). Comparatively fewer studies have examined the plasticity of CHCs in termites and the role they play in communication (reviewed in Bagnères & Hanus, 2015). CHC profiles may, in a given species, differ quantitatively and/or qualitatively among populations, colonies, castes, and seasons (Brent, Penick, Trobaugh, Moore, & Liebig, 2016; Darrouzet et al., 2014; Haverty, Grace, Nelson, & Yamamoto, 1996; Shelton & Grace, 1996; Shelton & Grace 1997). Several studies found a correlation between agonistic behaviors and differing CHC profiles by introducing intraspecific and interspecific groups with different chemical profiles (Bagnères, Killian, Clément, & Lange, 1991; Delphia, Copren, & Haverty, 2003; Haverty, Copren, Getty, & Lewis, 1999; Haverty, Page, Thorne, & Escoubas, 1991; Kaib et al., 2004; Takahashi & Gassa, 1995). At the same time, other studies failed to find a clear link between CHC profiles and agonism in termites (Chouvenc & Su, 2017; Su & Haverty, 1991), and it may not always be clear which compounds of the complex CHC profiles play a major role in the recognition processes. One potential explanation for this discrepancy is the relatively narrow window of a colony's lifespan in which CHCs in termites have been previously studied, with investigators potentially missing the effects of variability in CHC profiles over time, as recently suggested by the changes in chemical profiles of termite soldiers according to their age (Mitaka & Matsuura, 2020).

Incipient colonies of termites are subjected to different pressures compared to those from mature colonies. Firstly, incipient termite colonies (<1 year old) are under considerable developmental stress (Chouvenc, Basille, Li, & Su, 2014; Chouvenc, Basille, & Su, 2017) owing to a number of factors, resulting in suboptimal growth conditions including environmental fluctuations, limited resources, competition, disease pressure, and the variable amount of the parental investment (Chouvenc, 2019; Cole, Ilieş, & Rosengaus, 2018; Oster & Wilson, 1978). As a result, incipient colonies have a low success rate for their survival (Nutting, 1969). However, while the stress imposed on developing individuals within an incipient colony is relatively high (Chouvenc et al., 2014), it progressively decreases in older, more stable colonies, as newly produced individuals express a gradual reduction in developmental instability, while homeostatic conditions set within a maturing colony (Chouvenc, Scheffrahn, Mullins, & Su, 2017). Secondly, profiles of the recognition cues within a colony may differ by caste, sex, instar, or reproductive status (Clément & Bagnères, 1998; Funaro et al., 2018; Haverty et al., 1996; Howard et al., 1982; Howard & Blomquist, 1982, 2005), and the proportions of castes may differ based on seasons and on colony age (Howard & Haverty, 1981), including variation in the demographics of different‐aged worker instars (Chouvenc & Su, 2014). In incipient colonies, the king, queen, eggs, and larvae represent most of the initial colony biomass, while this ratio is shifted toward workers in older colonies. Finally, developmental pathways within colonies of *Coptotermes* and other “lower” termites are somewhat flexible and undergo changes that are directly related to colony age and demographics (Buchli, 1958; Chouvenc & Su, 2014; Hanus, Šobotník, Valterová, & Lukáš, 2006; Haverty, 1977). It is therefore possible that high developmental instability, reflected in individual morphologies, may also be expressed in their chemical signatures. If such high intracolonial CHC profile variability exists in young colonies, this may help to understand frequent fusions of young colonies observed in various termite species (Chouvenc & Su, 2017; Guaraldo & Costa‐Leonardo, 2009; Howard, Johns, Breisch, & Thorne, 2013; Korb, 2018), which can lead to colonies of mixed genetic structure (Vargo, 2019).

Owing to the difficulty of studying termite colonies over their life cycle, from incipient colony to maturity, no comprehensive research has investigated how CHC profiles of particular castes in the colony may change over the time. It is possible that such chemical changes may reflect the size or maturity status of a colony (Matsuura & Nishida, 2001). Additionally, changes in CHC profiles of individual castes within a colony over time are not represented in ant literature (Sprenger & Menzel, 2020), though several studies have found chemical profiles shift with age (Cuvillier‐Hot, Cobb, Malosse, & Peeters, 2001), reproductive status (Liebig, Peeters, Oldham, Markstadter, & Holldobler, 2000), and intracolonial contact (Bagnères & Morgan, 1991; Bonavita‐Cougourdan, Clément, & Lange, 1987; Corbara & Errard, 1991). Therefore, we focused on determining if the age of a termite colony is a relevant factor for the within‐colony variability of its inherent CHC profiles among castes in the Asian subterranean termite, *Coptotermes gestroi* (Wasmann, 1896; Blattodea: Rhinotermitidae), and discuss its potential ecological and evolutionary implications.

## 2 MATERIALS AND METHODS

### 2.1 Study insects and rearing conditions

We used colonies of *C. gestroi* established from alates collected during dispersal flights at a single location in Broward Co., FL, each year between 2015 and 2018. Incipient colonies were established as described by Chouvenc et al. (2014), by pairing a male and a female, reared in plastic cylindrical vials containing organic soil matter, wood, and 3% agarose. After one year, colonies were transferred to 1.5‐liter plastic boxes and two‐year‐old colonies were then transferred to 13‐liter plastic boxes. All rearing containers included organic soil, topsoil, and blocks of *Picea* sp. and were maintained at an average temperature of ~28°C, as in Chouvenc and Su (2017). The standardization of rearing conditions, diet, and the limited genetic variability among colonies (owing to *C. gestroi* having recently established in South Florida) minimized the effects of genetic factors that may have confounded colony age as the primary factor of focus in the current study. Previous observations on colony size (TC, personal observation) indicated that, at 6 months old, colonies consisted of less than 75 individuals, 18‐month‐old colonies contained ~1,000 individuals, 30‐month‐old colonies ~5,000 individuals and 42‐month‐old colonies approximately 30,000 individuals.

### 2.2 Sampling and chemical extraction and GC‐MS analysis

Termite colonies (*n* = 3 per age category) of 6, 18, 30, and 42 months were destructively sampled and processed caste by caste. Soldiers were excluded from the sampling as their frontal gland secretion extracted along with CHCs damages the chromatographic column (JMG, personal observation). Individuals (king and queen) or groups of individuals (workers and the brood) were stunned at −20°C for 5 min in clean glass Petri dishes before being extracted in hexane in 2‐ml vials. The queen and the king of each colony were individually extracted in 200 μl hexane. Groups of 50 workers randomly selected from each colony were used when available (some 6‐month‐old colonies had less than 50 workers, in which case all workers were used) and extracted in 200 μL hexane. Owing to their relatively small size and limited availability in incipient colonies, the brood (larvae and eggs) were all collected and pooled in a single sample for each colony of origin; however, because of the large brood in older colonies, the volume of hexane used for the brood extraction was proportional to the size of the brood (~50 μl per 20 mg of brood). Samples were vortexed for 30 min at the lowest setting for CHC elution (SI‐0236 Vortex‐Genie 2 variable speed vortexer with horizontal multi‐tube holder, USA Scientific, Ocala, Florida).

The hexane extracts were then transferred to 200 μl inserts inside of clean 2‐ml vials, concentrated to ~10 µl by solvent evaporation under mild N_2_ flow over 15 min, and 2 µl subsamples were manually injected into a gas chromatograph–mass spectrometer (GC‐MS) (Agilent 7890B/5977A Series Gas Chromatograph/Mass Selective Detector, Agilent Technologies, Santa Clara, California). The blends were separated by an HP‐5MS Ultra Inert column, 30 m × 250 μm × 0.25 μm (Agilent technologies 19091S‐433UI), using the following method: splitless inlet set initially at 290°C with a 2 ml/min flow of He carrying gas, oven temperature ramp from 60°C to 195°C at 20°C/min, then 195°C to 290°C at 3°C/min, and 290°C to 325°C at 15°C/min with a final 25 min hold time.

A standard solution of saturated hydrocarbons (C_4_ to C_40_) was used to obtain the retention times of compounds of interest in order to confirm their identity using their MS profiles. Compounds obtained from termite extracts with a primary structure between C_25_ and C_28_ carbons were considered in the analyses, as this range of CHCs dominates in *Coptotermes* cuticular waxes (Chouvenc & Su, 2017). Areas under GC peaks were integrated to estimate the raw abundance (mV^2^) of each peak. The retention time, in combination with the MS profile of each peak, was used to identify the compound size and determine the position of methyl groups in branched alkanes, using the 2014 edition of the National Institute of Standards and Technology (NIST) mass spectral library. Poor matches due to trace quantities of some minor compounds (≤C_24_ and ≥C_29_) were discarded from the outputs altogether. Due to the range of raw abundances across castes and colonies for some of the compounds of interest, the relative abundance of each CHC was used for comparison of samples.

### 2.3 CHC profiles analysis

A between‐class analysis (BCA, Culhane, Perriere, Considine, Cotter, & Higgins, 2002; Dolédec & Chessel, 1987; Thioulouse et al., 2018) was performed to understand differences of overall CHC profile variability between castes and ages. Cumulative overall abundance for the 13 CHC compounds of interest was used to determine the relative abundance of each CHC for all samples. Compounds were identified using spectral matching and those 13 that were reliably identified as hydrocarbons were included in this study. We thus compared the CHC abundance for each class of colony age (6, 18, 30, and 42 months) and caste (queens, kings, workers, and brood) alone and combined.

Overall changes in the intracolonial variability of CHC profiles over time were examined by using the coefficient of variation (CV) values of all independent CHCs. These caste‐wise, intracolonial chemical distance index values were calculated for each colony and a given CHC, by determining the variability (standard deviation) of the relative abundance of each given CHC across castes of a colony and dividing by the average relative abundance of each given CHC. A linear model was used to determine the relationship between the age of the colony and CV values, and for the overall changes in relative abundance of each independent alkane for all given castes. All statistical analyses were conducted in R version 3.3.1 (R Core Team, 2017). The package ade4 (Dray & Dufour, 2007) was used for the multivariate analyses (BCA).

## 3 RESULTS

### 3.1 CHC profiles in *Coptotermes gestroi*

**FIGURE 1:**
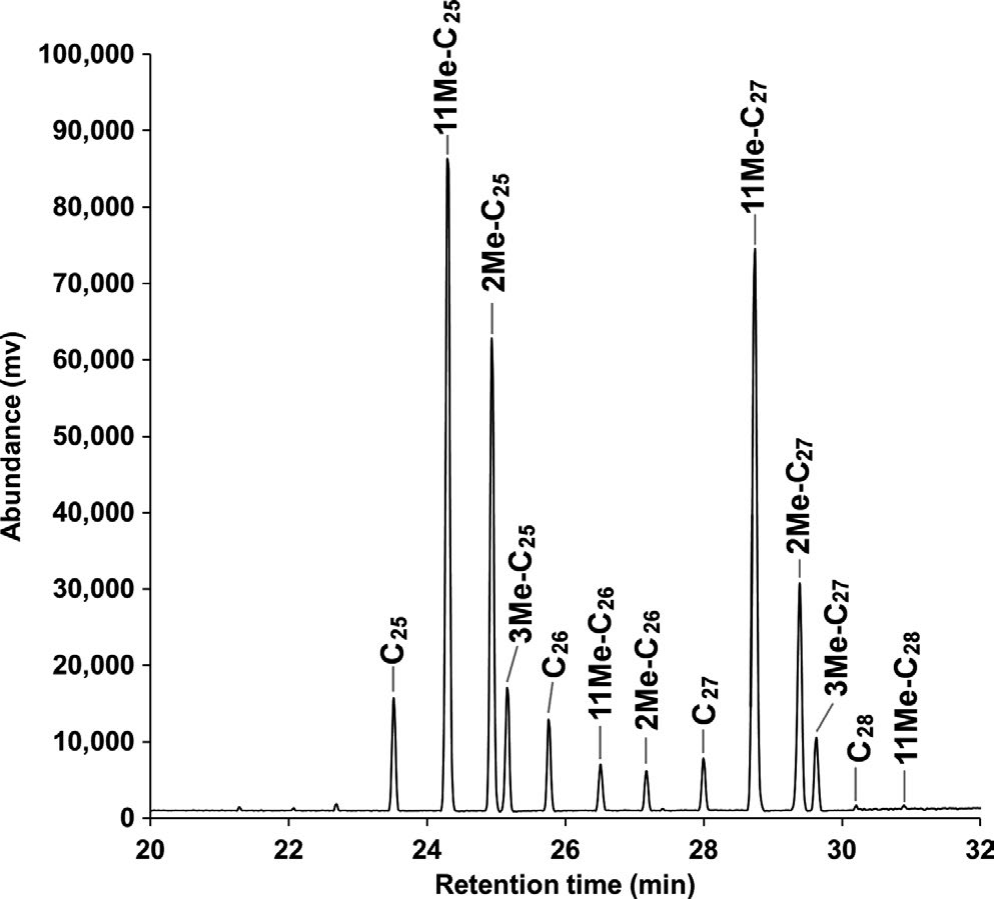
Chromatogram of the cuticular hydrocarbon (CHC) profile from workers in a 30‐month‐old *Coptotermes gestroi* colony. Each peak represents a compound (see Table 1 for abbreviations), and the areas under the peaks are indicative of their raw abundance

Thirteen CHC compounds were identified from all *C. gestroi* samples (Table 1), from C_25_ to C_28_, with the presence of methylated compounds at the 2nd, 3rd, and 11th positions. The relative abundances of each compound varied within and among caste and age of the colony, but 11Me‐C_25_ and 11Me‐C_27_ were the two most abundant CHCs found in the majority of samples (with an example of worker profile on Figure 1). No queen‐ or king‐specific CHC was identified in *C. gestroi* in this analysis, regardless of the age of the colony. An analysis of the intercolonial variability using a BCA showed that most colonies overlapped in their overall chemical profiles (Appendix S1), indicating colony of origin was not a contributing factor for the overall variability of the chemical profiles.

**TABLE 1:** Cuticular hydrocarbons from *Coptotermes gestroi* identified and quantified from gas chromatography–mass spectrometry

Abbreviation	Full name	Compound structure	Average relative proportion across all castes and colony age classes (Mean ± *SE*)
C_25_	Pentacosane	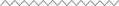	9.03 ± 1.67%
11Me‐C_25_	11‐Methyl Pentacosane	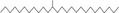	22.51 ± 1.05%
2Me‐C_25_	2‐Methyl Pentacosane	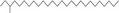	17.29 ± 1.25%
3Me‐C_25_	3‐Methyl Pentacosane	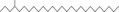	2.94 ± 0.21%
C_26_	Hexacosane	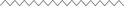	3.31 ± 0.40%
11Me‐C_26_	11‐Methyl Hexacosane	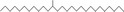	1.47 ± 0.17%
2Me‐C_26_	2‐Methyl Hexacosane	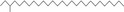	1.60 ± 0.18%
C_27_	Heptacosane	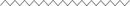	3.82 ± 0.60%
11Me‐C_27_	11‐Methyl Heptacosane	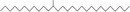	25.71 ± 1.37%
2Me‐_C27_	2‐Methyl Heptacosane	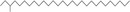	9.70 ± 1.00%
3Me‐_C27_	3‐Methyl Heptacosane	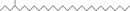	2.11 ± 0.19%
C28	Octacosane	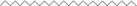	0.16 ± 0.03%
11Me‐C_28_	11‐Methyl Octacosane	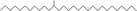	0.34 ± 0.05%

### 3.2 Caste‐wise differences in CHC profiles in *Coptotermes gestroi*

When focusing solely on intercaste CHC variability independently of the age of the colony, the difference among four castes studied (workers, brood, king, and queen) accounted for 36% of the total variance. The first axis of the BCA explained a large part of the difference (74%), while the following axes were less important (15% for the second, 11% the third, and others negligible). The CHC profiles of the kings and queens were distinct from those of the brood, but with a small overlap with workers (Appendix S3).

### 3.3 Colony age‐wise differences in CHC profiles in *Coptotermes gestroi*

The CHC variability among colony age classes independent of caste variability accounted for 20% of the total variance. The first two axes of the BCA explained almost the same part of the difference (48% and 43%, respectively), the third axis was less important (8%), and further ones negligible, accounting for 1% altogether. Age classes were partially distinct from one another (Appendix S2): The two intermediate age classes (18 and 30 months) largely overlapped, while the youngest and oldest age classes were clearly distinct.

### 3.4 Interaction of age and caste in differences in CHC profiles in *Coptotermes gestroi*

**FIGURE 2:**
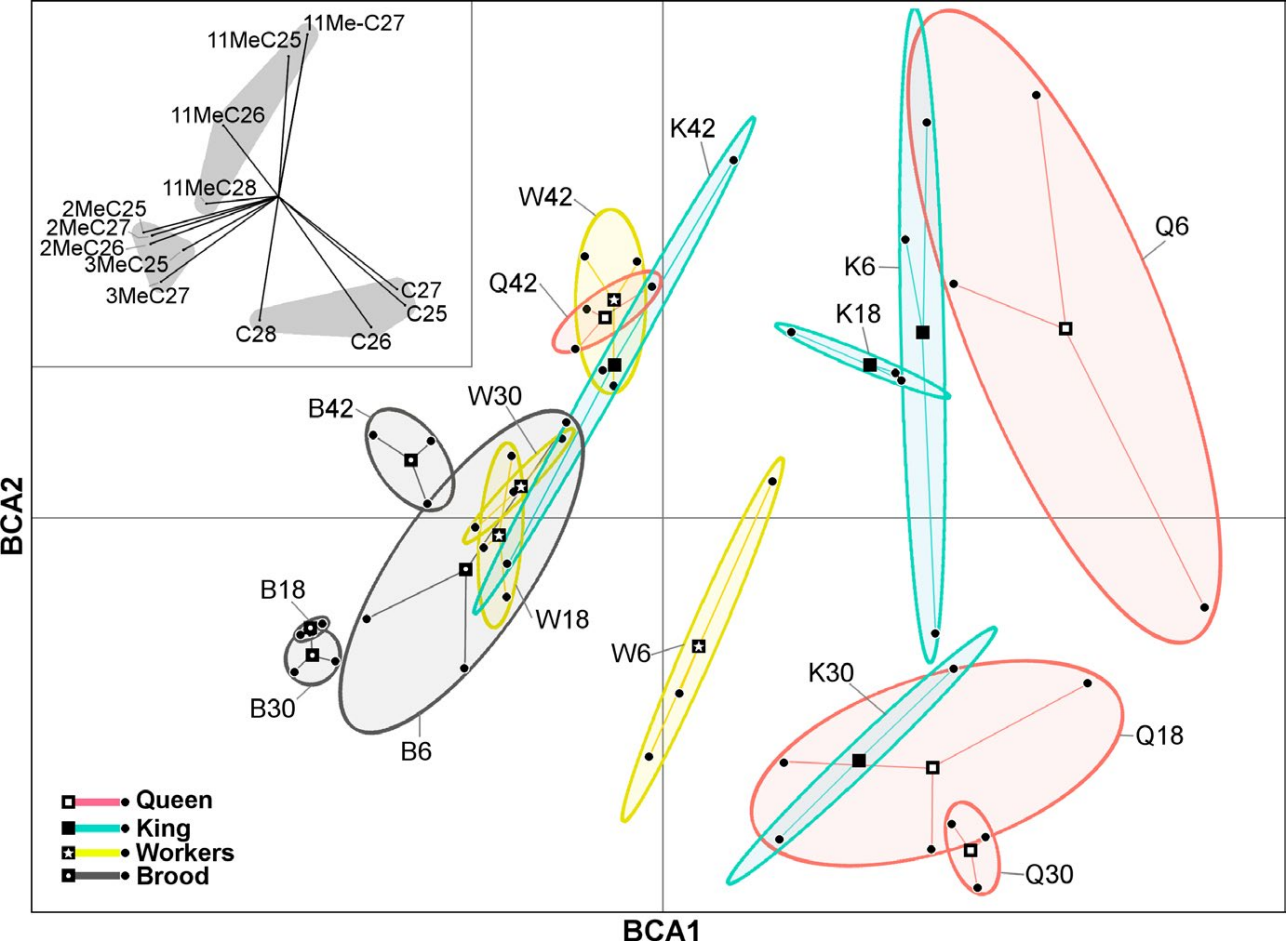
Between‐class analysis (BCA) of termite extract samples for 13 cuticular hydrocarbons (identified and quantified as in Figure 1), taking into account the interaction between two factors: colony age and caste, in *Coptotermes gestroi*. Each ellipse represents the scatter of the interaction between caste and colony age, with letters on labels: Q = queen, K = king, W = workers, and B = brood (eggs + larvae), and values on labels: 6 = 6‐month‐old colonies, 18 = 18‐month‐old colonies, 30 = 30‐month‐old colonies, and 42 = 42‐month‐old colonies (i.e., K30 represent kings from 30‐month‐old colonies, with *n* = 3 per ellipse). Vectors indicate the effect each of cuticular hydrocarbon (CHC) on the analysis, with gray area representing levels of correlations among CHCs

When examining the variability of CHC profiles using the interaction of the two factors (age of the colony and the caste), the BCA had a higher explanatory power on the observed variance than the two factors independently, as the difference between groups accounted for 77% of the total variance (first axis: 25%; second axis: 14%; third axis: 9%; fourth axis: 3%; fifth axis: 2%; and each of the other axes < 1%). The multivariate analysis showed that all castes had distinct profiles in young colonies, but by 42 months, all castes had converged toward a similar CHC profile (Figure 2). Concerning particular CHC compounds, it was mostly the increase in relative abundance of methylated alkanes (accompanied by the decrease in linear CHCs) that discriminated between age classes (Appendices S4–S7), and all castes at 42 months tended to converge toward a more homogenous profile (Figure 2). Finally, the brood displayed remarkably limited variability in CHC profiles, regardless of the age of the colony.

### 3.5 Changes in chemical profiles over time

In spite of a high variability in CHC profiles among castes, colonies, and age classes, some particular compounds displayed significant changes in relative abundance over time as shown by linear models (Appendices S4–S7). C_25_ in the brood (*p* = .015; Appendix S4) and both C_25_ and C_26_ in workers (*p* < .01; *p* < .01; Appendix S5) proportionally decreased as colonies aged, while both 11Me‐C_26_ and 11Me‐C_27_ proportionally increased over time in workers (*p* < .01; *p* < .01; Appendix S5) and the brood (*p* = .0104;* p* < .01; Appendix S4), with 11Me‐C_27_ being particularly dominant in both castes. 2Me‐C_25_ and 2Me‐C_26_ proportionally increased over time in reproductives, kings (*p* = .0132; *p* = .0156; Appendix S6), and queens (*p* < .01; *p* < .01; Appendix S7), and 2Me‐C_27_ and 3Me‐C_27_ proportionally increased over time in queens only (*p* < .01; *p* < .01; Appendix S7). Several other trends in proportional CHC changes within castes were observed, but owing to the relatively low number of replicates, no other significant relationships were found for other compounds over time in any given castes.

**FIGURE 3:**
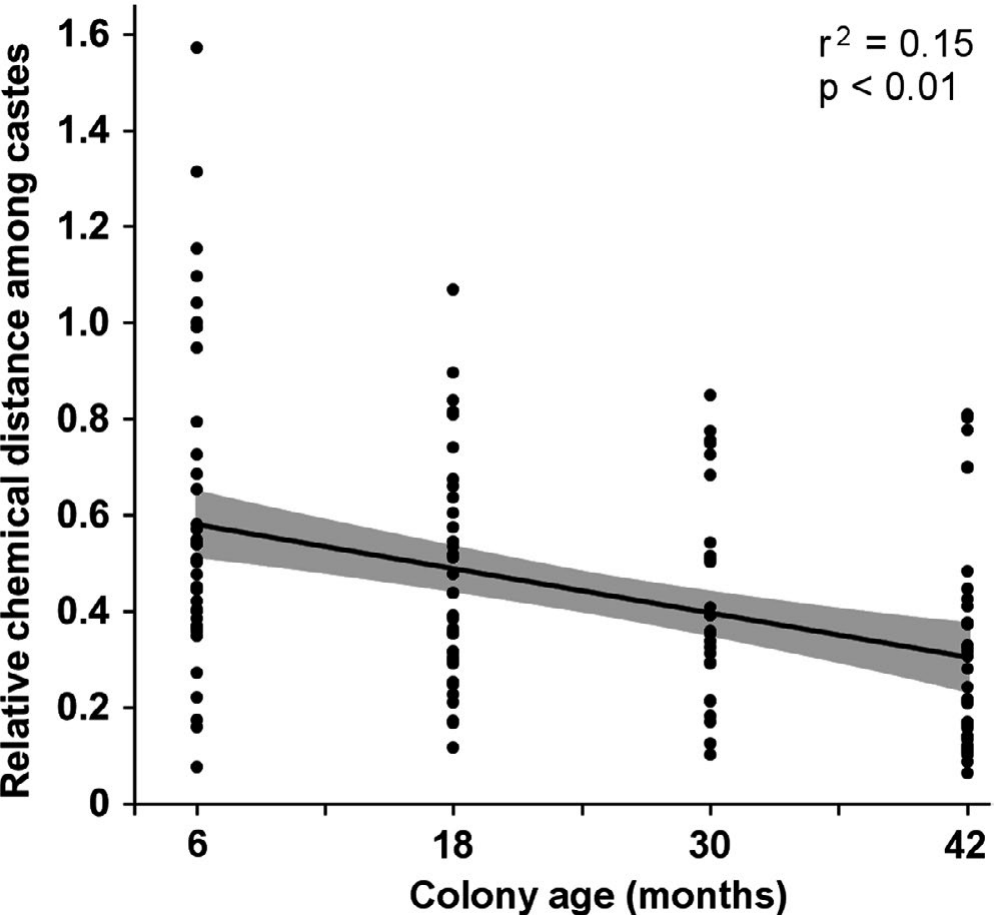
Changes in coefficient of variation (CV) values over time, indicating within‐colony change in relative chemical distance among castes over time. Each data point represents the CV value of one compound among all castes within a single *Coptotermes gestroi* colony. The shaded gray area represents the 95% confidence interval of the linear model

Finally, the coefficient of variation (CV) values showed that intracolonial chemical variability decreased with age (*p < *.01; Figure 3). Young colonies displayed relatively high chemical heterogeneity in their CHC profiles among castes, and this heterogeneity progressively decreased as colonies aged, with all caste‐specific profiles converging over time toward a more similar intracolonial CHC profile, confirming the results obtained from BCA analysis in Figure 2.

## 4 DISCUSSION

Our results show that the cuticular hydrocarbon profile of *Coptotermes gestroi* consisted of a blend of 13 dominant compounds in all castes and colony age classes, with 11Me‐C_25_ and 11Me‐C_27_ dominant in most of the samples. This confirms the findings of Chouvenc and Su (2017), although we herein focused on the most abundant compounds only. Though the overall qualitative CHC profiles remained unchanged across all samples, the relative representation of particular CHC species varied, from trace to dominant compound, among castes, and across colony age classes. This result shows that both the caste and age of a termite colony are relevant factors influencing the CHC profiles of individual termites. We showed that the intracolonial variability in CHC profiles was much higher in young colonies compared to older, and the high heterogeneity of chemical profiles within young colonies progressively reduces as the colony ages. In 6‐month‐old colonies, the CHC profiles were distinct from each other in all studied castes (queen, king, workers, and brood), and the caste‐specific CHC profiles converged as the colony matured. In other words, the reproductives and brood become, over time, more similar to workers, the most abundant caste in large colonies (roughly 80% of the biomass in 42‐month‐old colonies, TC, personal observation). In addition, the overall changes in the CHC profile of workers over time may be influenced by the change in demographic composition of colonies, as older worker instars accumulate in older colonies (Chouvenc & Su, 2014).

The CHC profile of *Coptotermes* soldiers could not be determined in this study because of the copious amounts of the frontal gland secretion (up to 36% of living weight of soldier; Waller & La Fage, 1987), which unavoidably contaminated the extracts. Our preliminary tests showed that such secretions were problematic in the GC‐MS, damaging the chromatographic columns. Haverty et al. (1990) indicated that soldiers’ CHC profile in *C. formosanus* usually matches the workers’ CHC profile, qualitatively; however, these results were not submitted to rigorous statistical testing. In the light of our results, the similarity in CHC profiles between the two castes (see Haverty et al., 1990) could be the result of the relative homogeneity of the colony scent across castes found in mature colonies. In retrospect, it therefore seems likely that the first few soldiers in incipient colonies, which express extreme forms of developmental instability (Chouvenc et al., 2014), may also produce variable CHC profiles, distinct from all other castes, potentially increasing the initial intracolonial chemical heterogeneity. Such CHC variability in soldiers still needs to be confirmed, and a different extraction protocol, such as solid‐phase microextraction (SPME), should be tested in the future.

A constant habituation to changing chemical profiles has been suggested in ants, in which colony scent plasticity was repeatedly observed (Isingrini, Lenoir, & Jaisson, 1985; Vander Meer et al., 1989; Wallis, 1963). This concept may also apply to termites, with some evidence for flexible recognition of nestmates over time (Lee, Mullins, Aguilera‐Olivares, Chouvenc, & Su, 2019). Additionally, under the *Gestalt‐odor* model (Crozier & Dix, 1979), often used to explain nestmate recognition in social Hymenoptera, individual profiles of members of the colony are pooled via grooming, trophallaxis, and other physical contacts among nestmates, resulting in a relatively uniform colony odor, but are still subject to ongoing quantitative changes caused by variations from environmental factors (Lenoir et al., 2001). Termites may use CHCs to establish a colony profile in the same way (Marten, Kaib, & Brandl, 2010), as the colony gestalt for CHCs is progressively established as a colony matures. However, the brood was the only group within the analysis that displayed minimal variation over time. This observation may be explained by a couple of factors: Firstly, the eggshell and thin cuticle of the larvae have cuticle than may not be fully capable of producing the wide range of CHC observed in workers, potentially resulting in a simple and conserved CHC profile, comprised of the few major components displayed by the species. Secondly, a simplified CHC profile may be the result of the brood being protected in the central part of the nest where most environmental variations are buffered with minimal interferences, where they interact primarily with early instar workers (Du, Chouvenc, Osbrink, & Su, 2017). Thirdly, uniform profile of larvae might be adaptive, protecting them from agonism if two colonies merge or the weaker one is conquered by a stronger one (Lee et al., 2019). Therefore, our study showed that the CHC profile of termites changes at two distinct time scale: age of the colony and the age of each individual (*i.e.,* instar stage). However, our observations also implied that there may be a confounding factor in the overall CHC profile of workers, beyond the age the colony, as mature colonies accumulate older worker instars (4th, 5th, and 6th worker instars), while younger worker instars (up to the 3rd) dominate in incipient colonies. The same is true for soldiers that differentiate from younger instars in incipient colonies (Buchli, 1958; Chouvenc & Su, 2014; Hanus et al., 2006) and may thus also contribute to the overall variability of the cuticular signatures. Similarly, the emergence of nymphs and alates in fully mature colonies could also be a factor driving the dynamic chemical profile of a termite colony. Likewise, prior to alate flights, the proportion of soldiers in the colony increases in order to provide protection to swarming alates (Haverty et al., 1996; Howard & Haverty, 1981). Such seasonal changes in caste proportions may ultimately alter overall colony scent as well, likely with respect to quantity of compounds present (Haverty et al., 1990). The potential roles in CHC profile changes of these variables (older worker and soldier instars and alate production) were not addressed in the current study as we were limited to the destructive sampling of colonies up to 42 months old. Therefore, future research should investigate such potential additional contributions toward the understanding of the dynamics of CHC colony profile in termites in older colonies.

With the observation of the relatively high quantitative variability in CHC profile in young termite colonies, we here argue that this leads to a poorly defined colony scent, resulting in habituation to a relatively broad spectrum of CHC profiles recognized as nestmates, which does not interfere with the detection of qualitative differences among termite species (Howard & Blomquist, 1982, 2005). Intraspecific fusion of incipient colonies without overt agonism among workers and soldiers (Chouvenc & Su, 2017; Guaraldo & Costa‐Leonardo, ; Howard et al., 2013; Korb, 2018; Lee et al., 2019) can be explained as a consequence of “plastic acceptance threshold” (Bagnères & Hanus, 2015; Reeve, 1989). With the relatively high intracolonial dissimilarity of CHC profiles in young colonies, the threshold for the acceptance of a potential conspecific non‐nestmate would therefore be relatively high. However, the acceptance threshold likely decreases over time as the overall colony odor converges, and the colony members probably become habituated to a narrowed colony scent profile and would thus become more sensitive to CHC profile dissimilarities.

Our results support the presence of a high tolerance threshold for CHC profile dissimilarity in incipient termite colonies that may have deep ecological and evolutionary implications. High CHC variability in younger incipient colonies can result in a poorly defined colony gestalt, which may temporarily and passively alter how such a tolerance threshold is established, at a time when colonies are under high stress and vulnerable to intraspecific competition from surrounding colonies, including incipient ones, established during large dispersal flight events (Chouvenc, Scheffrahn, et al., 2017; Nutting, 1969; Thorne, Breisch, & Muscedere, 2003). Because incipient termite colonies have a small margin of error for initial colony survival (Chouvenc, 2019; Chouvenc & Su, 2017; Nutting, 1969) systematic aggression from the recognition of non‐nestmates in incipient colonies may therefore result in death and failure of both incipient colonies. As such, young colonies with high acceptance thresholds would survive the encounter through colony merging. While such an event may result in a relatively reduced relatedness among individuals in a colony, at least temporarily, a peaceful merging of workers, soldiers, and brood of the intraspecific colonies may be more beneficial for the overall fitness of the newly formed mixed family (Lee et al., 2019; Vargo, 2019). In contrast, an immediate agonistic interaction among soldiers and workers could result in mutual destruction (Matsuura & Nishida, 2001). This may be especially true for incipient colonies early in their development, which cannot afford to lose the few individuals they have during agonistic confrontations. The occurrence of such young colonies merging has been used to support the “accelerated inheritance” hypothesis (Thorne et al., 2003) as a possible pathway for the initial evolution of eusociality in termites. This hypothesis, which proposes that elder juveniles would inherit the breeding position and the existing workforce in the event of the loss of primary reproductives and, in the context of intraspecific competition among incipient colonies, has been suggested as having been a driving factor for the initial emergence of the soldier caste, ultimately toward eusociality (Thorne et al., 2003). This hypothesis has been debated (Korb & Roux, 2012), but convincingly been argued against, as all aspects of eusociality would have had to be acquired prior to the emergence of such inheritance events (Bignell, 2016; Nalepa, 2015; Roisin, 2015). However, in the light of our current study, the observations by Thorne et al. (2003), Johns, Howard, Breisch, Rivera, and Thorne (2009), Guaraldo and Costa‐Leonardo (), and Howard et al. (2013) all reveal the proximal mechanism by which young termite colonies merge: Workers and soldiers in such colonies may have a relatively high acceptance threshold with a potentially poor ability to recognize non‐nestmates owing to variable chemical profiles in their environment. With such observations, we here argue that, throughout the radiation and the evolution of extant termite species, no selective pressure has driven incipient colonies to readily exclude intraspecific non‐nestmates. Indeed, for a lack of agonism among intraspecific incipient termite colonies to be maintained over evolutionary times, such a trait had to be positively selected for or neutral; otherwise, some mechanisms would have evolved to prevent such early colony fusion. In the first scenario of colony merging, even if one of the royal pair is eventually eliminated (Guaraldo & Costa‐Leonardo, ; Thorne et al., 2003), each genetic lineage would still have a 50% chance of survival (neutral selection), and the surviving lineage would inherit the rest of the working force (positive selection). In the second scenario, strong agonism would most likely result in mutual destruction for both incipient colony (negative selection). Our results therefore suggest that the merging of conspecific incipient colonies is a passive process resulting from the potential absence of non‐nestmate recognition.

To conclude, this study revealed the variable profile of CHCs in *Coptotermes gestroi*, among castes and over colony development, and that the overall intracolonial scent homogenizes over time. High stress in incipient colonies results in a temporal window in which a high acceptance threshold for non‐nestmates, in place due to high variability and lack of distinct chemical profiles of younger colonies, could potentially result in avoidance of mutual destruction for both colonies in an interaction and fusion event. Such CHC variability in incipient colonies may be widespread in extant termites and we suggest that colonies with high acceptance thresholds may have been subjected to either neutral or positive selection, depending on the intraspecies competition conditions, which has allowed for the observed fusion of incipient colonies. Additionally, the reduction in variability of CHC profiles over time, as colony size increases, may explain the incongruities observed in termite recognition mechanisms, with some observations showing high agonism levels among conspecific colonies (for review see Shelton & Grace, 1996; Thorne & Haverty, 1991) and others demonstrating high prevalence of colony fusion events (Chouvenc & Su, 2017; Vargo, 2019; Vargo & Husseneder, 2011).

## CONFLICT OF INTEREST

None Declared.

## AUTHOR CONTRIBUTIONS

**Johnalyn M. Gordon:** Conceptualization (equal); Data curation (lead); Formal analysis (equal); Funding acquisition (supporting); Investigation (lead); Methodology (equal); Project administration (lead); Resources (supporting); Software (equal); Supervision (equal); Validation (lead); Visualization (equal); Writing‐original draft (lead); Writing‐review & editing (equal). **Jan Šobotnik:** Conceptualization (equal); Data curation (supporting); Formal analysis (supporting); Funding acquisition (supporting); Investigation (supporting); Methodology (supporting); Project administration (supporting); Visualization (equal); Writing‐review & editing (equal). **Thomas Chouvenc:** Conceptualization (equal); Data curation (supporting); Formal analysis (equal); Funding acquisition (lead); Investigation (supporting); Methodology (equal); Project administration (supporting); Resources (lead); Software (equal); Supervision (equal); Validation (equal); Visualization (equal); Writing‐review & editing (equal).

## Supporting information

Supplementary Material

## Data Availability

Relative cuticular hydrocarbon abundances: Dryad https://doi.org/10.5061/dryad.59zw3r24h

## References

[ece36669-bib-0001] Bagnères, A.‐G. , & Hanus, R. (2015). Communication and social regulation in termites Social Recognition in Invertebrates, 1, 193–248. 10.1007/978-3-319-17599-7_11

[ece36669-bib-0002] Bagnères, A.‐G. , Killian, A. , Clément, J.‐L. , & Lange, C. (1991). Interspecific recognition among termites of the genus *Reticulitermes*: Evidence for a role for the cuticular hydrocarbons. Journal of Chemical Ecology, 17(12), 2397–2420. 10.1007/BF00994590 24258635

[ece36669-bib-0003] Bagnères, A.‐G. , & Morgan, E. D. (1991). The postpharyngeal glands and the cuticle of Formicidae contain the same characteristic hydrocarbons. Experientia, 47, 106–111. 10.1007/BF02041269

[ece36669-bib-0004] Bagnères, A.‐G. , Rivière, G. , & Clément, J.‐L. (1998). Artificial neural network modeling of caste odor discrimination based on cuticular hydrocarbons in termites. Chemoecology, 8(4), 201–209. 10.1007/s000490050026

[ece36669-bib-0005] Bignell, D. E. (2016). The role of symbionts in the evolution of termites and their rise to ecological dominance in the tropics In Hurst C. (Ed.), The mechanistic benefits of microbial symbionts, advances in environmental microbiology (Vol. 2, pp. 121–172). Cham, Switzerland: Springer.

[ece36669-bib-0006] Blomquist, G. J. , Nelson, D. R. , & Renobales, M. D. (1987). Chemistry, biochemistry, and physiology of insect cuticular lipids. Archives of Insect Biochemistry and Physiology, 6(4), 227–265. 10.1002/arch.940060404

[ece36669-bib-0007] Bonavita‐Cougourdan, A. , Clément, J. L. , & Lange, C. (1987). Nestmate recognition: the role of cuticular hydrocarbons in the ant *Camponotus vagus* Scop. Journal of Entomological Science, 22, 1–10. 10.18474/0749-8004-22.1.1

[ece36669-bib-0008] Brent, C. S. , Penick, C. A. , Trobaugh, B. , Moore, D. , & Liebig, J. (2016). Induction of a reproductive‐specific cuticular hydrocarbon profile by a juvenile hormone analog in the termite *Zootermopsis nevadensis* . Chemoecology, 26(5), 195–203. 10.1007/s00049-016-0219-8

[ece36669-bib-0009] Buchli, H. (1958). L'origine des castes et les potentialités ontogéniques des Termites européens du genre *Reticulitermes* Holmgren. Annales Des Sciences Naturelles, Zoologie, 20, 263–429.

[ece36669-bib-0010] Chapman, R. F. (2012). Integument In Simpson S. J., & Douglas A. E. (Eds.), The insects: Structure and function (5th ed., pp. 415–438). New York, NY: Cambridge University Press.

[ece36669-bib-0011] Chouvenc, T. (2019). The relative importance of queen and king initial weights in termite colony foundation success. Insectes Sociaux, 66(2), 177–184. 10.1007/s00040-019-00690-3

[ece36669-bib-0012] Chouvenc, T. , Basille, M. , Li, H.‐F. , & Su, N.‐Y. (2014). Developmental instability in incipient colonies of social insects. PLoS One, 9(11), e113949 10.1371/journal.pone.0113949 25423502 PMC4244189

[ece36669-bib-0013] Chouvenc, T. , Basille, M. , & Su, N.‐Y. (2017). Role of accelerated developmental pathway and limited nurturing capacity on soldier developmental instability in subterranean termite incipient colonies. Insectes Sociaux, 64(4), 477–483. 10.1007/s00040-017-0566-7

[ece36669-bib-0014] Chouvenc, T. , Scheffrahn, R. H. , Mullins, A. J. , & Su, N.‐Y. (2017). Flight phenology of two *Coptotermes* species (Isoptera: Rhinotermitidae) in Southeastern Florida. Journal of Economic Entomology, 110(4), 1693–1704. 10.1093/jee/tox136 28854645

[ece36669-bib-0016] Chouvenc, T. , & Su, N.‐Y. (2014). Colony age‐dependent pathway in caste development of *Coptotermes formosanus* Shiraki. Insectes Sociaux, 61(2), 171–182. 10.1007/s00040-014-0343-9

[ece36669-bib-0017] Chouvenc, T. , & Su, N.‐Y. (2017). Testing the role of cuticular hydrocarbons on intercolonial agonism in two subterranean termite species (*Coptotermes*) and their hybrids. Insectes Sociaux, 64(3), 347–355. 10.1007/s00040-017-0552-0

[ece36669-bib-0018] Clément, J.‐L. (1982). Signaux de contact responsables de l’agression interspécifique des Termites du genre *Reticulitermes* (Isoptères). Comptes Rendus Des Seances De L'academie Des Sciences, 294(12), 635–638.

[ece36669-bib-0019] Clément, J.‐L. , & Bagnères, A.‐G. (1998). Nestmate recognition in termites In Vander Meer R. K. (Ed.), Pheromone communication in social insects: Ants, wasps, bees, and termites (pp. 126–155). Boulder, CO: Westview Press.

[ece36669-bib-0020] Cole, E. L. , Ilieş, I. , & Rosengaus, R. B. (2018). Competing physiological demands during incipient colony foundation in a social insect: Consequences of pathogenic stress. Frontiers in Ecology and Evolution, 6, 1–12. 10.3389/fevo.2018.00103

[ece36669-bib-0021] Corbara, B. , & Errard, C. (1991). The organization of artificial heterospecific ant colonies. The case of the *Manica rubida*/*Formica selys*i association: Mixed colony or parallel colonies? Behavioural Processes, 23, 75–87. 24923201 10.1016/0376-6357(91)90107-B

[ece36669-bib-0022] Crozier, R. H. , & Dix, M. W. (1979). Analysis of two genetic models for the innate components of colony odor in social Hymenoptera. Behavioral Ecology and Sociobiology, 4(3), 217–224.

[ece36669-bib-0023] Culhane, A. , Perriere, G. , Considine, E. , Cotter, T. , & Higgins, D. (2002). Between‐group analysis of microarray data. Bioinformatics, 18(12), 1600–1608. 10.1093/bioinformatics/18.12.1600 12490444

[ece36669-bib-0024] Cuvillier‐Hot, V. , Cobb, M. , Malosse, C. , & Peeters, C. (2001). Sex, age and ovarian activity affect cuticular hydrocarbons in *Diacamma ceylonense*, a queenless ant. Journal of Insect Physiology, 47, 485–493. 10.1016/S0022-1910(00)00137-2 11166313

[ece36669-bib-0025] Dani, F. R. , Jones, G. R. , Corsi, S. , Beard, R. , Pradella, D. , & Turillazzi, S. (2005). Nestmate recognition cues in the honey bee: Differential importance of cuticular alkanes and alkenes. Chemical Senses, 30(6), 477–489. 10.1093/chemse/bji040 15917370

[ece36669-bib-0080] Darrouzet, E. , Labédan, M. , Landré, X. , Perdereau, E. , Christidès, J.P. , Bagnères, A.G. (2014). Endocrine control of cuticular hydrocarbon profiles during worker‐to‐soldier differentiation in the termite *Reticulitermes flavipes* . Journal of Insect Physiology, 61, 25–33. 10.1016/j.jinsphys.2013.12.006 24374106

[ece36669-bib-0026] Delphia, C. M. , Copren, K. A. , & Haverty, M. I. (2003). Agonistic behavior between individual worker termites from three cuticular hydrocarbon phenotypes of *Reticulitermes* (Isoptera: Rhinotermitidae) from Northern California. Annals of the Entomological Society of America, 96(4), 585–593.

[ece36669-bib-0027] Dettner, K. (2014). Chemical ecology and biochemistry of dytiscidae In Yee D. (Ed.), Ecology, systematics, and the natural history of predaceous diving beetles (Coleoptera: Dytiscidae) (pp. 235–306). Dordrecht, the Netherlands: Springer.

[ece36669-bib-0028] Dolédec, S. , & Chessel, D. (1987). Rythmes saisonniers et composantes stationnelles en milieu aquatique. I‐ Description d’un plan d’observations complet par projection de variables. Acta Oecol Oecol Generalis, 8(3), 403–426.

[ece36669-bib-0029] Dray, S. , & Dufour, A.‐B. (2007). Theade4Package: Implementing the duality diagram for ecologists. Journal of Statistical Software, 22(4), 1–20.

[ece36669-bib-0030] Du, H. , Chouvenc, T. , Osbrink, W. L. A. , & Su, N. Y. (2017). Heterogeneous distribution of castes/instars and behaviors in the nest of *Coptotermes formosanus* Shiraki. Insectes Sociaux, 64(1), 103–112.

[ece36669-bib-0081] Funaro, C. F. , Böröczky, K. , Vargo, E. L. , Schal, C. (2018). Identification of a queen and king recognition pheromone in the subterranean termite *Reticulitermes flavipes* . Proceedings of the National Academy of Sciences, 115(15), 3888–3893. 10.1073/pnas.1721419115 PMC5899469 29555778

[ece36669-bib-0031] Guaraldo, A. C. , & Costa‐Leonardo, A. M. (2009). Preliminary fusion testing between whole young colonies of *Coptotermes gestroi* (Isoptera: Rhinotermitidae). Sociobiology, 53(3), 767–774.

[ece36669-bib-0032] Guerrieri, F. J. , Nehring, V. , Jørgensen, C. G. , Nielsen, J. , Galizia, C. G. , & D’Ettorre, P. (2009). Ants recognize foes and not friends. Proceedings of the Royal Society B: Biological Sciences, 276(1666), 2461–2468. 10.1098/rspb.2008.1860 PMC2690455 19364750

[ece36669-bib-0033] Hanus, R. , Šobotník, J. , Valterová, I. , & Lukáš, J. (2006). The ontogeny of soldiers in *Prorhinotermes simplex* (Isoptera, Rhinotermitidae). Insectes Sociaux, 53, 249–257.

[ece36669-bib-0034] Haverty, M. I. (1977). The proportion of soldiers in termite colonies: A list and a bibliography (Isoptera). Sociobiology, 2(3), 199–216.

[ece36669-bib-0035] Haverty, M. I. , Copren, K. A. , Getty, G. M. , & Lewis, V. R. (1999). Agonistic behavior and cuticular hydrocarbon phenotypes of colonies of reticulitermes (Isoptera: Rhinotermitidae) from Northern California. Annals of the Entomological Society of America, 92(2), 269–277. 10.1093/aesa/92.2.269

[ece36669-bib-0036] Haverty, M. I. , Grace, J. K. , Nelson, L. J. , & Yamamoto, R. T. (1996). Intercaste, intercolony, and temporal variation in cuticular hydrocarbons of *Coptotermes formosanus* Shiraki (Isoptera: Rhinotermitidae). Journal of Chemical Ecology, 22(10), 1813–1834. 24227110 10.1007/BF02028506

[ece36669-bib-0037] Haverty, M. I. , Nelson, L. J. , & Page, M. (1990). Cuticular hydrocarbons of four populations of *Coptotermes formosanus* Shiraki in the United States. Journal of Chemical Ecology, 16(5), 1635–1647. 10.1007/BF01014096 24263833

[ece36669-bib-0038] Haverty, M. I. , Page, M. , Thorne, B. , & Escoubas, P. (1991). Cuticular hydrocarbons: Species and population‐level discrimination in termites. USDA Forest Service – General Technical Report. PSW‐128, 15–23.

[ece36669-bib-0039] Howard, K. J. , Johns, P. M. , Breisch, N. L. , & Thorne, B. L. (2013). Frequent colony fusions provide opportunities for helpers to become reproductives in the termite *Zootermopsis nevadensis* . Behavioral Ecology and Sociobiology, 67(10), 1575–1585. 10.1007/s00265-013-1569-7

[ece36669-bib-0040] Howard, R. W. , & Blomquist, G. J. (1982). Chemical ecology and biochemistry of insect hydrocarbons. Annual Review of Entomology, 27, 149–172. 10.1146/annurev-ento-031620-071754 33417824

[ece36669-bib-0041] Howard, R. W. , & Blomquist, G. J. (2005). Ecological, behavioral, and biochemical aspects of insect hydrocarbons. Annual Review of Entomology, 50(1), 371–393. 10.1146/annurev.ento.50.071803.130359 15355247

[ece36669-bib-0042] Howard, R. W. , & Haverty, M. I. (1981). Seasonal variation in caste proportions of field colonies of *Reticulitermes flavipes* (Kollar). Environmental Entomology, 10(4), 546–549.

[ece36669-bib-0043] Howard, R. W. , McDaniel, C. A. , & Blomquist, G. J. (1978). Cuticular hydrocarbons of the eastern subterranean termite, *Reticulitermes flavipes* (Kollar) (Isoptera: Rhinotermitidae). Journal of Chemical Ecology, 4(2), 233–245. 10.1007/BF00988058

[ece36669-bib-0044] Howard, R. W. , McDaniel, C. A. , & Blomquist, G. J. (1980). Chemical mimicry as an integrating mechanism: Cuticular hydrocarbons of a termitophile and its host. Science, 210(4468), 431–433. 17837424 10.1126/science.210.4468.431

[ece36669-bib-0045] Howard, R. W. , McDaniel, C. A. , Nelson, D. R. , Blomquist, G. J. , Gelbaum, L. T. , & Zalkow, L. H. (1982). Cuticular hydrocarbons of *Reticulitermes virginicus* (Banks) and their role as potential species‐ and caste‐recognition cues. Journal of Chemical Ecology, 8(9), 1227–1239. 10.1007/BF00990755 24413965

[ece36669-bib-0046] Isingrini, M. , Lenoir, A. , & Jaisson, P. (1985). Preimaginal learning as a basis of colony‐brood recognition in the ant *Cataglyphis cursor* . Proceedings of the National Academy of Sciences of the United States of America, 82(24), 8545–8547. 10.1073/pnas.82.24.8545 16593637 PMC390953

[ece36669-bib-0047] Johns, P. M. , Howard, K. J. , Breisch, N. L. , Rivera, A. , & Thorne, B. L. (2009). Nonrelatives inherit colony resources in a primitive termite. Proceedings of the National Academy of Sciences of the United States of America, 106(41), 17452–17456. 10.1073/pnas.0907961106 19805058 PMC2757400

[ece36669-bib-0048] Kaib, M. , Jmhasly, P. , Wilfert, L. , Durka, W. , Franke, S. , Francke, W. , … Brandl, R. (2004). Cuticular hydrocarbons and aggression in the termite *Macrotermes subhyalinus* . Journal of Chemical Ecology, 30(2), 365–385. 10.1023/B:JOEC.0000017983.89279.c5 15112730

[ece36669-bib-0049] Korb, J. (2018). Chemical fertility signaling in termites: Idiosyncrasies and commonalities in comparison with ants. Journal of Chemical Ecology, 44(9), 818–826. 10.1007/s10886-018-0952-2 29616376

[ece36669-bib-0050] Korb, J. , & Roux, E. A. (2012). Why join a neighbour: Fitness consequences of colony fusions in termites. Journal of Evolutionary Biology, 25(11), 2161–2170. 10.1111/j.1420-9101.2012.02617.x 22998731

[ece36669-bib-0051] Lee, S.‐B. , Mullins, A. , Aguilera‐Olivares, D. , Chouvenc, T. , & Su, N.‐Y. (2019). Fused colonies of the formosan subterranean termite (Blattodea: Rhinotermitidae) for laboratory experiments. Journal of Economic Entomology, 112(5), 2311–2315. 10.1093/jee/toz154 31165146

[ece36669-bib-0052] Lenoir, A. , D'Ettorre, P. , Errard, C. , & Hefetz, A. (2001). Chemical ecology and social parasitism in ants. Annual Review of Entomology, 46, 573–599. 10.1146/annurev.ento.46.1.573 11112180

[ece36669-bib-0053] Liebig, J. , Peeters, C. , Oldham, N. J. , Markstadter, C. , & Holldobler, B. (2000). Are variations in cuticular hydrocarbons of queens and workers a reliable signal of fertility in the ant *Harpegnathos saltator*? Proceedings of the National Academy of Sciences of the United States of America, 97, 4124–4131. 10.1073/pnas.97.8.4124 10760282 PMC18170

[ece36669-bib-0054] Marten, A. , Kaib, M. , & Brandl, R. (2010). Are cuticular hydrocarbons involved in speciation of fungus‐growing termites (Isoptera: Macrotermitinae)? In Glaubrecht M. (Ed.), Evolution in action (pp. 283–306). Berlin, Heidelberg, Germany: Springer.

[ece36669-bib-0055] Martin, S. , & Drijfhout, F. (2009). A review of ant cuticular hydrocarbons. Journal of Chemical Ecology, 35(10), 1151–1161. 10.1007/s10886-009-9695-4 19866237

[ece36669-bib-0056] Matsuura, K. , & Nishida, T. (2001). Colony fusion in a termite: What makes the society "open"? Insectes Sociaux, 48, 378–383. 10.1007/PL00001795

[ece36669-bib-0057] Mitaka, Y. , & Matsuura, K. (2020). Age‐dependent increase in soldier pheromone of the termite *Reticulitermes speratus* . Journal of Chemical Ecology, 46(5–6), 483–489. 10.1007/s10886-020-01182-6 32440722

[ece36669-bib-0058] Nalepa, C. A. (2015). Origin of termite eusociality: Trophallaxis integrates the social, nutritional, and microbial environments. Ecological Entomology, 40(4), 323–335. 10.1111/een.12197

[ece36669-bib-0060] Nutting, W. L. (1969). Flight and colony foundation In Krishna K., & Weesner F. M. (Eds.), Biology of termites (Vol. 1, pp. 233–282). New York, NY: Academic Press.

[ece36669-bib-0061] Oster, G. F. , & Wilson, E. O. (1978). Caste and ecology in the social insects. Princeton, NJ: Princeton University Press. 740003

[ece36669-bib-0062] R Core Team . (2017). R: A language and environment for statistical computing. Vienna, Austria: R Foundation for Statistical Computing Retrieved from https://www.R-project.org/

[ece36669-bib-0063] Reeve, H. K. (1989). The evolution of conspecific acceptance thresholds. The American Naturalist, 133(3), 407–435. 10.1086/284926

[ece36669-bib-0064] Roisin, Y. (2015). What makes the cost of brood care important for the evolution of termite sociality? Its insignificance. Ecological Entomology, 41(1), 31–33. 10.1111/een.12278

[ece36669-bib-0065] Shelton, T. G. , & Grace, J. K. (1996). Review of agonistic behaviors in the Isoptera. Sociobiology, 28(2), 155–176.

[ece36669-bib-0066] Shelton, T. G. , & Grace, J. K. (1997). Suggestion of an environmental influence on intercolony agonism of Formosan subterranean termites (Isoptera: Rhinotermitidae). Environmental Entomology, 26(3), 632–637. 10.1093/ee/26.3.632

[ece36669-bib-0067] Singer, T. L. (1998). Roles of hydrocarbons in the recognition systems of insects. American Zoologist, 38(2), 394–405. 10.1093/icb/38.2.394

[ece36669-bib-0068] Sprenger, P. P. , & Menzel, F. (2020). Cuticular hydrocarbons in ants (Hymenoptera: Formicidae) and other insects: How and why they differ among individuals, colonies, and species. Myrmecological News, 30, 1–26.

[ece36669-bib-0069] Su, N.‐Y. , & Haverty, M. I. (1991). Agonistic behavior among colonies of the Formosan subterranean termite, *Coptotermes formosanus* Shiraki (Isoptera: Rhinotermitidae), from Florida and Hawaii: Lack of correlation with cuticular hydrocarbon composition. Journal of Insect Behavior, 4(1), 115–128. 10.1007/BF01092555

[ece36669-bib-0070] Takahashi, S. , & Gassa, A. (1995). Roles of cuticular hydrocarbons in intra‐and interspecific recognition behavior of two Rhinotermitidae species. Journal of Chemical Ecology, 21(11), 1837–1845. 10.1007/BF02033680 24233833

[ece36669-bib-0071] Thioulouse, J. , Dray, S. , Dufour, A.‐B. , Siberchicot, A. , Jombart, T. , & Pavoine, S. (2018). Multivariate analysis of ecological data with ade4. New York, NY: Springer.

[ece36669-bib-0072] Thorne, B. L. , Breisch, N. L. , & Muscedere, M. L. (2003). Evolution of eusociality and the soldier caste in termites: Influence of intraspecific competition and accelerated inheritance. Proceedings of the National Academy of Sciences of the United States of America, 100(22), 12808–12813. 10.1073/pnas.2133530100 14555764 PMC240700

[ece36669-bib-0073] Thorne, B. L. , & Haverty, M. I. (1991). A review of intracolony, intraspecific, and interspecific agonism in termites. Sociobiology, 19, 115–145.

[ece36669-bib-0074] van Zweden, J. S. , & D’Ettorre, P. (2010). Nestmate recognition in social insects and the role of hydrocarbons In Blomquist G. J., & Bagnères A.‐G. (Eds.), Insect hydrocarbons: Biology, biochemistry, and chemical ecology (pp. 222–243). Cambridge, UK: Cambridge University Press.

[ece36669-bib-0075] Vander Meer, R. K. , Saliwanchik, D. , & Lavine, B. (1989). Temporal changes in colony cuticular hydrocarbon patterns of *Solenopsis invicta* . Journal of Chemical Ecology, 15, 2115–2125. 10.1007/BF01207442 24272300

[ece36669-bib-0076] Vargo, E. L. (2019). Diversity of termite breeding systems. Insects, 10(2), 52 10.3390/insects10020052 PMC6409762 30759735

[ece36669-bib-0077] Vargo, E. L. , & Husseneder, C. (2011). Genetic structure of termite colonies In Bignell D. E., Roisin Y., & Lo N. (Eds.), Biology of termites: A modern synthesis (pp. 321–346). New York, NY: Springer.

[ece36669-bib-0078] Waller, D. A. , & La Fage, J. P. (1987). Unpalatability as a passive defense of *Coptotermes formosanus* Shiraki soldiers against ant predation. Journal of Applied Entomology, 103(1–5), 148–153.

[ece36669-bib-0079] Wallis, D. (1963). A comparison of the response to aggressive behaviour in two species of ants, *Formica fusca* and *Formica sanguinea* . Animal Behaviour, 11(1), 164–171. 10.1016/0003-3472(63)90025-3

