# Supplementary material for: Colony‐age‐dependent variation in cuticular hydrocarbon profiles in subterranean termite colonies
Source: Ecol Evol. 2020 Aug 16;10(18):10095–104. doi: 10.1002/ece3.6669 (PMC7520186; doi:10.1002/ece3.6669)
Supplement: Supplementary file 1 — Supplementary Material [file ECE3-10-10095-s001.docx]

**Appendix Materials**

for

**Colony-age-dependent variation in cuticular hydrocarbon profiles in subterranean termite colonies**

**Johnalyn M. Gordon, Jan Šobotník, and Thomas Chouvenc**

**
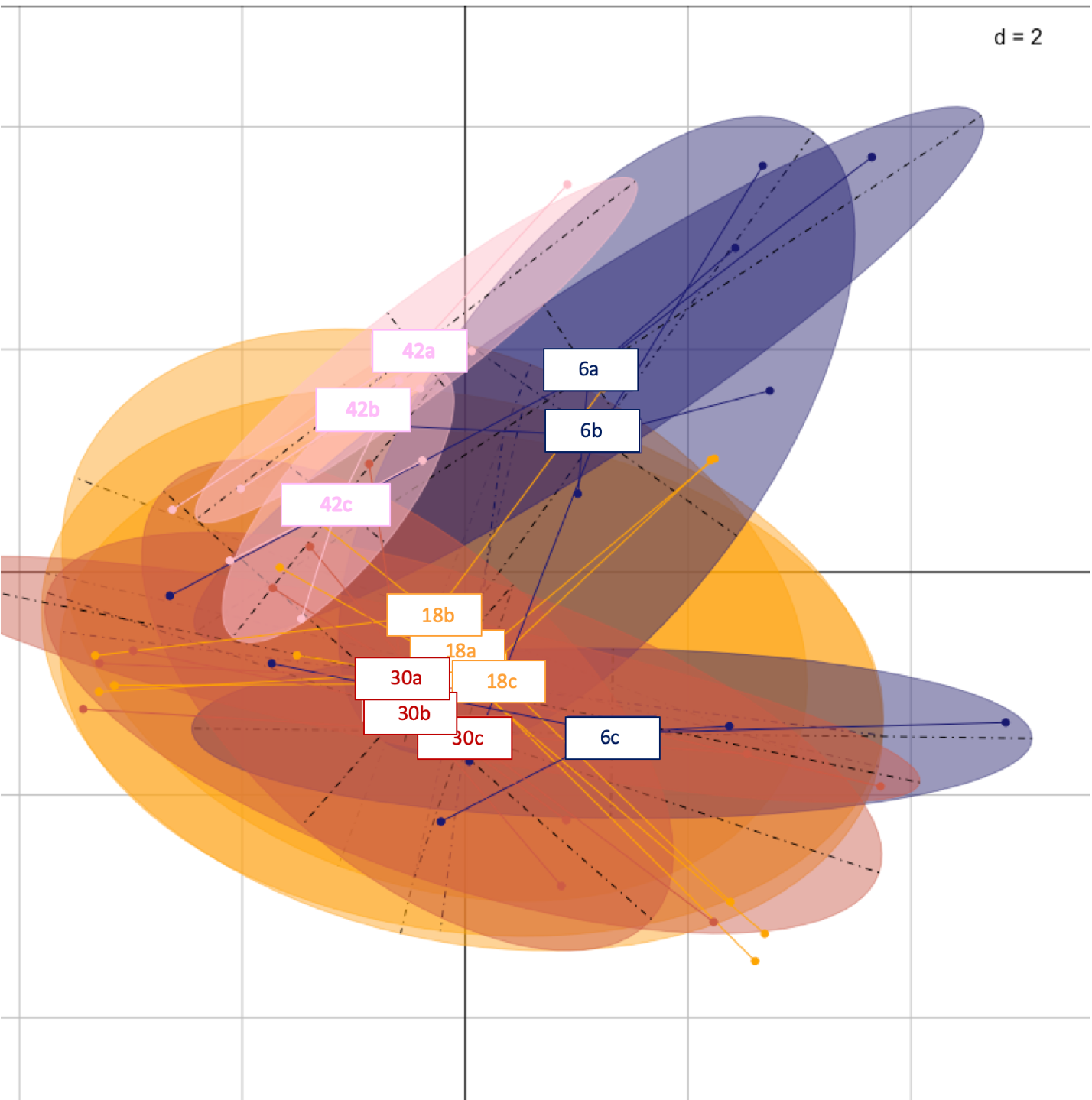
**

**Figure S1:** Between-class analysis of differences between colonies of *Coptotermes gestroi* at four difference age classes on the first two axes of the analysis. Different colonies at each age class (n=3) are represented alphabetically, as “a”, “b”, and “c”. Interaction groupings are encircled by ellipses.

**
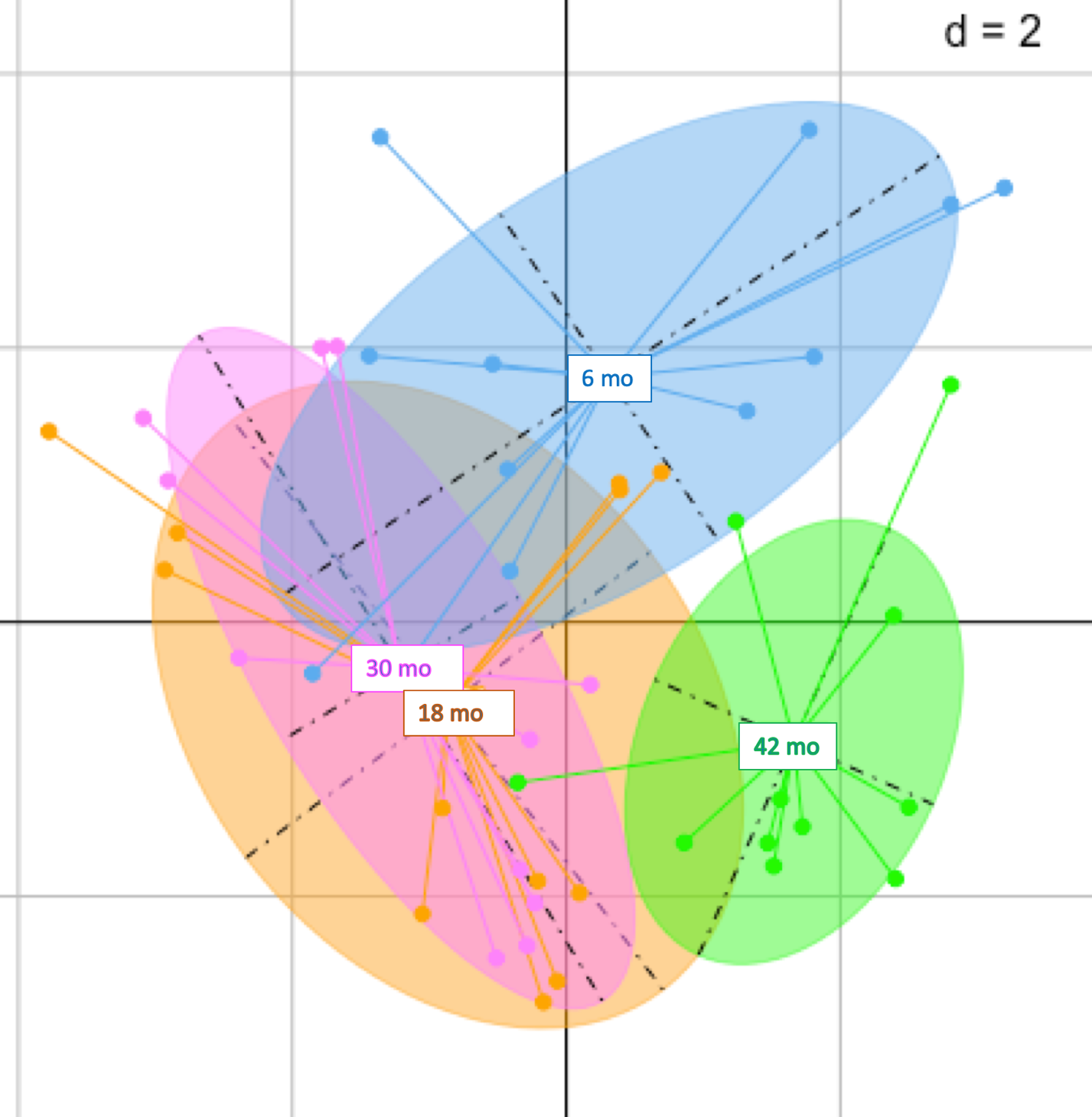
**

**Figure S2:** Age-specific between-class analysis of 13 cuticular hydrocarbons (CHCs) from colonies of *Coptotermes gestroi* on the first two axes of the analysis*.* Visualization of the age factor with 6-month-old, 18-month-old, 30-month-old, and 42-month-old age groupings encircled by ellipses.

**
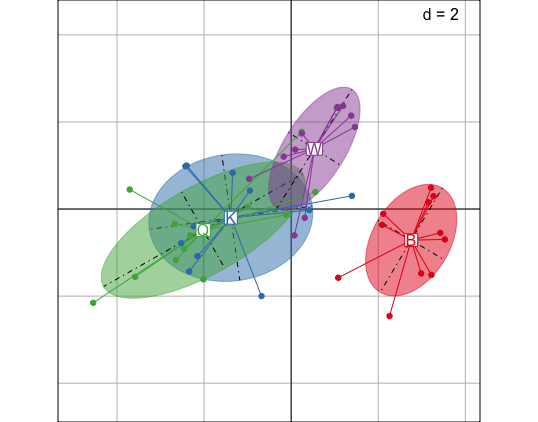
**

**Figure S3:** Caste-specific between-class analysis of 13 cuticular hydrocarbons (CHCs) from colonies of *Coptotermes gestroi* on the first two axes of the analysis*.* Visualization of the caste factor with King (K), Queen (Q), Workers (W), and Brood (B) groupings encircled by ellipses.


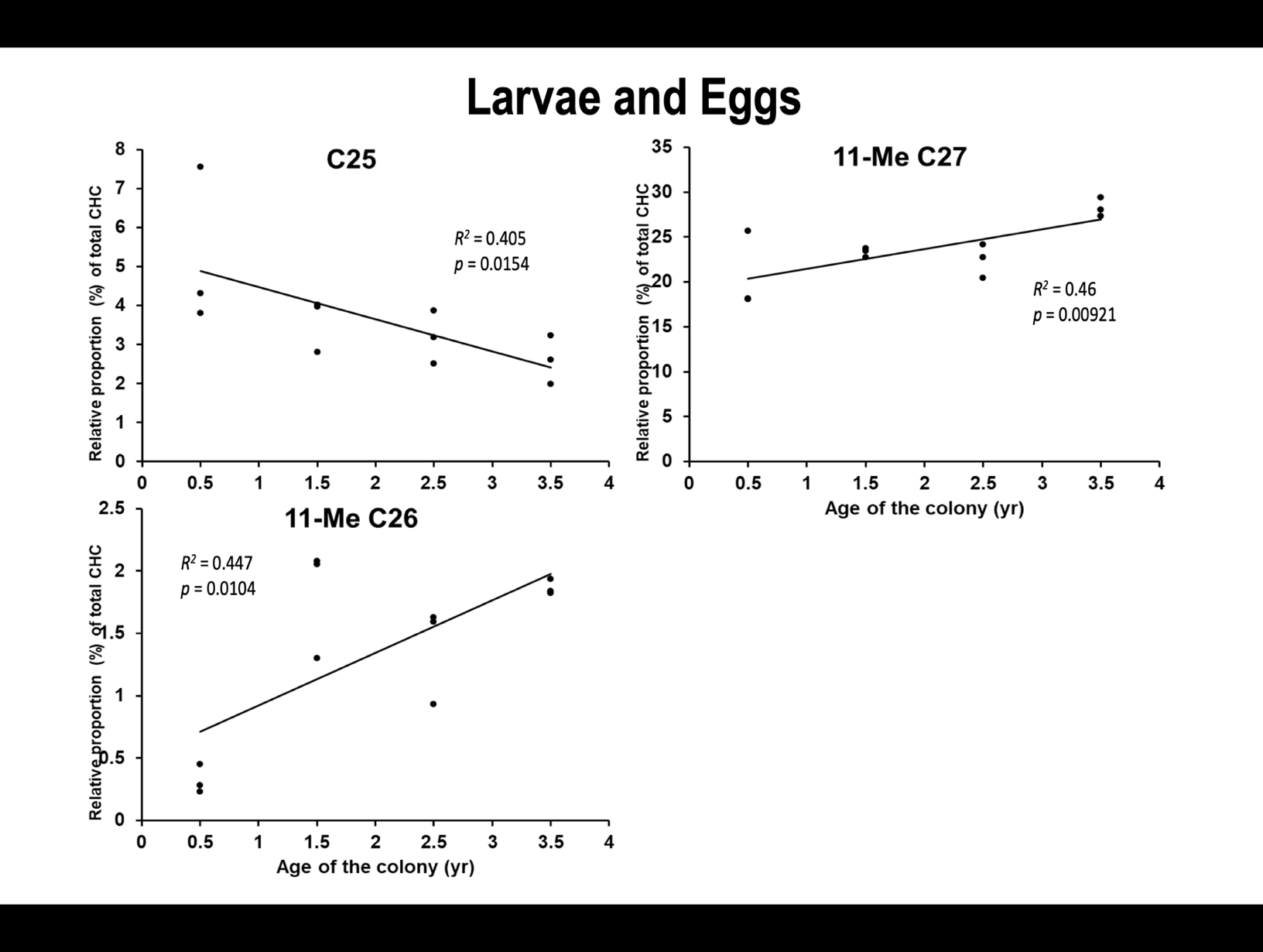


**Figure S4, brood.** Changes in the relative proportion of cuticular hydrocarbons (CHCs) over time in the brood. The figure shows 3 distinct CHCs with significant correlations (linear model) in relative abundance over time (yr). Each point indicates the caste sample from a colony.


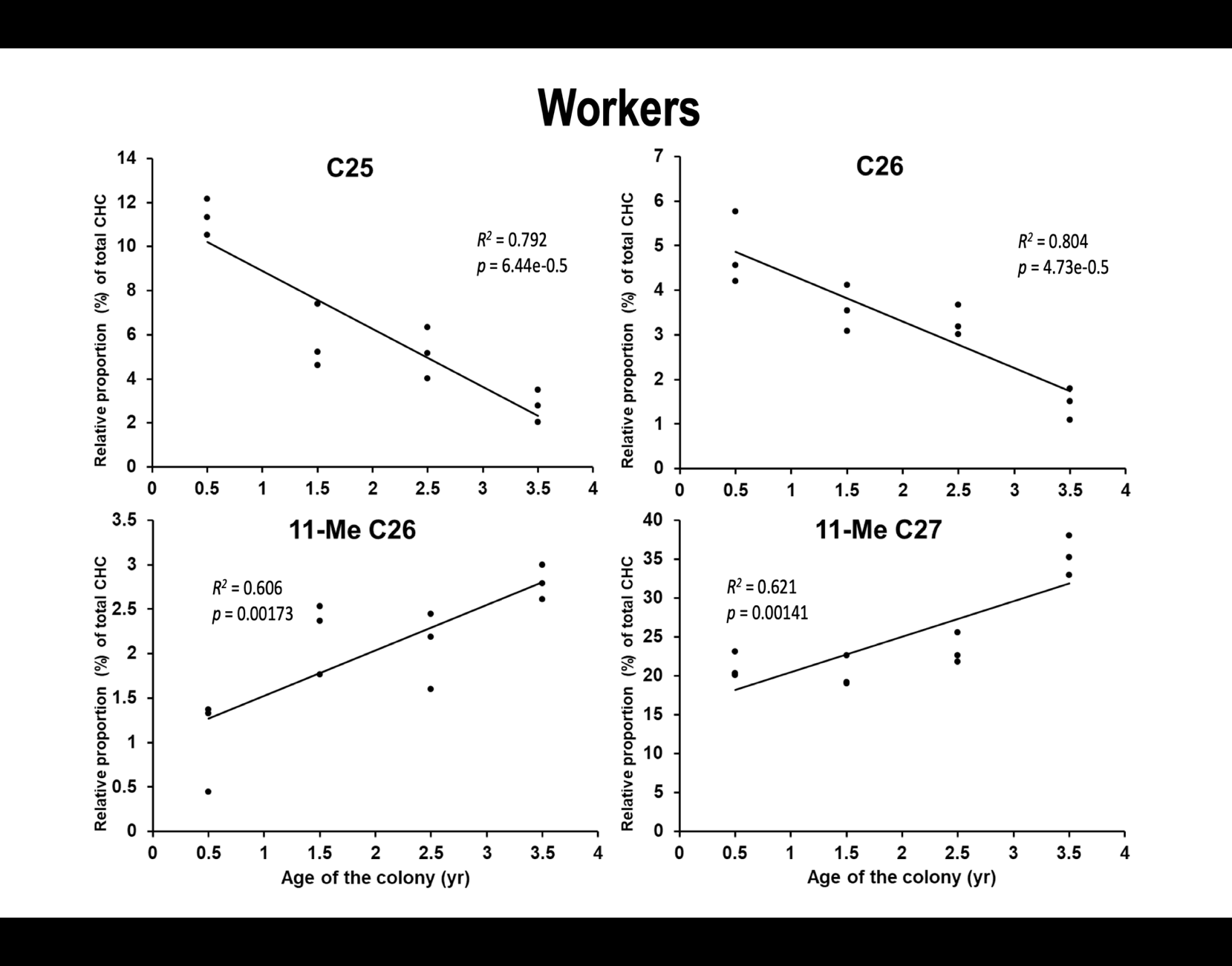


**Figure S5, workers.** Changes in the relative proportion of cuticular hydrocarbons (CHCs) over time in workers. The figure shows 3 distinct CHCs with significant correlations (linear model) in relative abundance over time (yr). Each point indicates the caste sample from a colony.


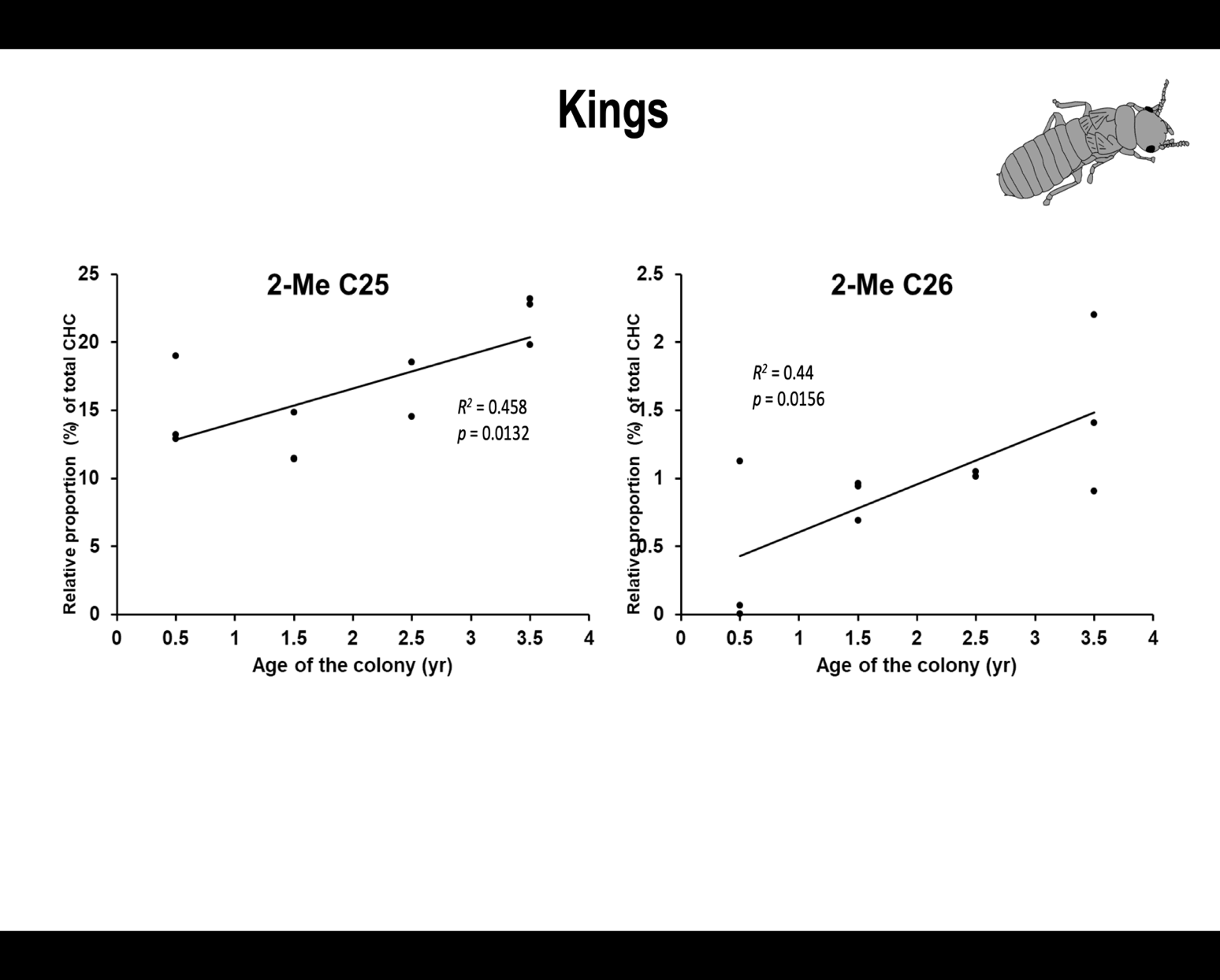


**Figure S6, king.** Changes in the relative proportion of cuticular hydrocarbons (CHCs) over time in kings. The figure shows 3 distinct CHCs with significant correlations (linear model) in relative abundance over time (yr). Each point indicates the caste sample from a colony.


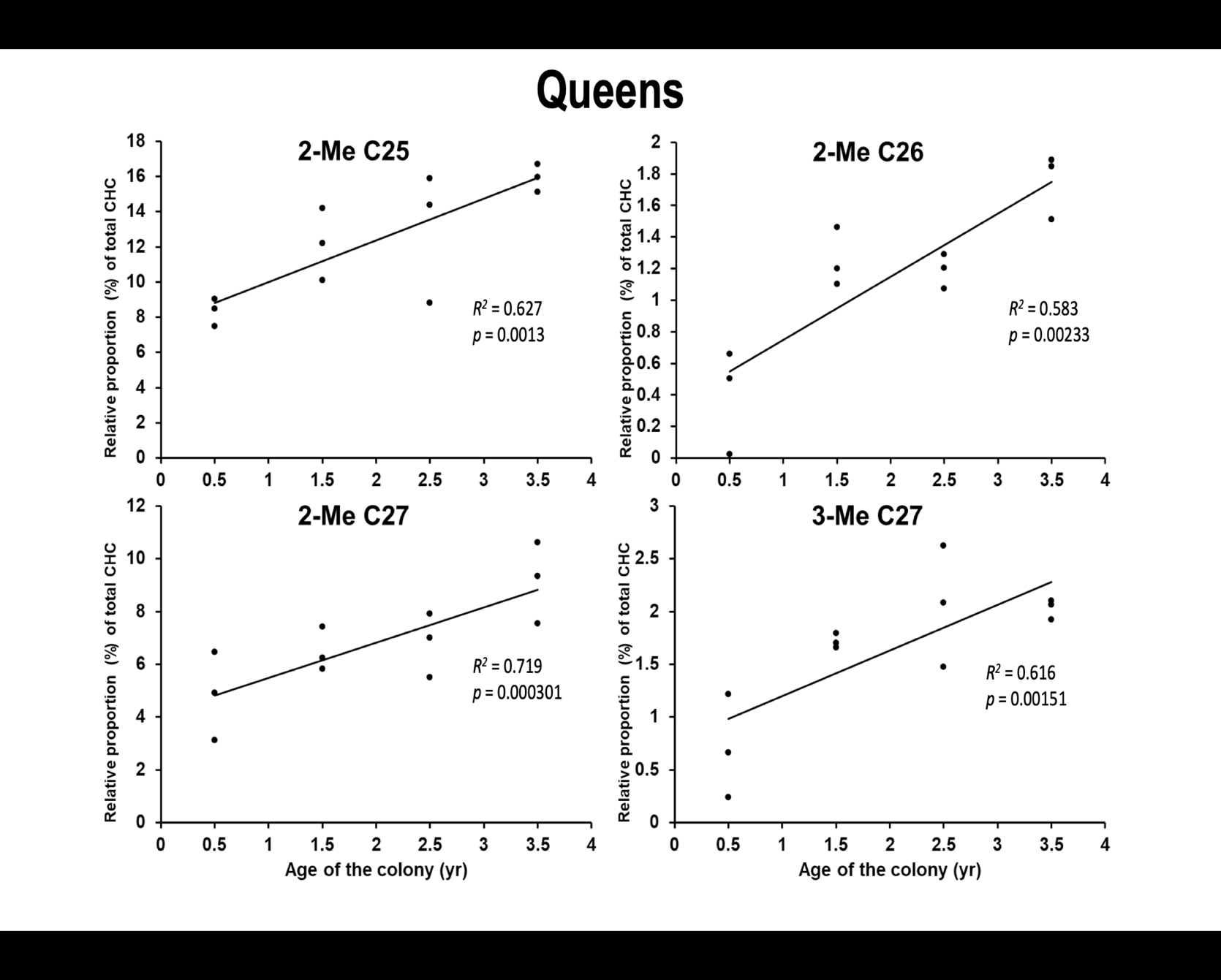


**Figure S7, queen.** Changes in the relative proportion of cuticular hydrocarbons (CHCs) over time in queens. The figure shows 3 distinct CHCs with significant correlations (linear model) in relative abundance over time (yr). Each point indicates the caste sample from a colony.
